# RP-HPTLC Retention Data in Correlation with the *In-silico* ADME Properties of a Series of s-triazine Derivatives

**Published:** 2014

**Authors:** Lidija R Jevrić, Sanja O Podunavac-Kuzmanović, Jaroslava V Švarc-Gajić, Strahinja Z Kovačević, Bratislav Ž Jovanović

**Affiliations:** a Department of Applied and Engineering Chemistry, Faculty of Technology, University of Novi Sad, Serbia.; b Institute for Chemistry, Technology and Metallurgy, University of Belgrade, Serbia.

**Keywords:** s-triazine derivatives, *In-silico*, ADME, PCA, Polynomial regression

## Abstract

The properties relevant to pharmacokinetics and pharmacodynamics of four series of synthesized *s*-triazine derivatives have been studied by Quantitative structure-retention relationship (QSRR) approach. The chromatographic behavior of these compounds was investigated by using reversed-phase high performance thin-layer chromatography (RP-HPTLC). Chromatographic retention (*R*_M_^0^) was correlated with selected physicochemical parameters relevant to pharmacokinetics, *i.e*. ADME (absorption, distribution, metabolism and excretion). In addition, the ability to act as kinase inhibitors and protease inhibitors was predicted for all investigated triazine classes. Also, in order to confirm similarities/dissimilarities between series of examined compounds, principal component analysis (PCA) based on calculated ADME properties was conducted. The *R*_M_^0^ values of the *s*-triazine derivatives have been recommended for description and evaluation of pharmacokinetic properties. According to results of this study, the synthesized s-triazine derivatives meet pharmacokinetic criteria of preselection for drug candidates.

## Introduction

Traditional drug development includes compound synthesis and pre-clinical *in-vitro* and *in-vivo* studies to determine whether such a compound can be considered as a candidate for clinical trial. Such procedures are normally accompanied with enormous costs measured in billions of dollars and more than a decade of interdisciplinary endeavor. In addition to high investments, many of tested candidates in later stages of drug development might demonstrate lack of efficiency, poor pharmacokinetics, animal toxicity and adverse effects in humans. For this, modern process of drug development is based on combinatorial chemistry, genomics, chemometrics and *in-silico* processing. While one group of these computational methods focuses on biological activity, trying to forecast interactions with target receptors (toxicodynamic), others tend to predict the fate of the substance in the human body *i.e*. its absorption, distribution, metabolism and excretion (ADME). Chemometrics has an important place in relating structural or property descriptors of a drug candidate to its biological activity (QSAR - Quantitative structure-activity relationship). 

The 1,3,5-triazine (*s*-triazine) heterocyclic system is today found in a number of bioactive molecules such as herbicides and pharmaceutical products (1). Various triazine substituted molecules exhibit diverse biological activities, having thus been reported as potentially cardiotonic (2,3), anti-HIV (4,5), antitumor (6) and anticancer agents (7). 

*s*-Triazine is a weak base with six-membered heterocyclic ring containing three nitrogens replacing carbon-hydrogen units in the benzene ring. The compound, so as its derivatives, has an excellent potential for the formation of non-covalent bonds, such as coordination and H-bonds, via its nitrogen ion-pairs (8). Non-covalent bonds have a very important role in biological activity of these compounds (9), but also in understanding of their physiological behavior, namely absorption, metabolism and elimination. Furthermore, such chemical properties of *s*-triazines are responsible for their characteristic chromatographic behaviour.

Molecular lipophilicity is one of the major physicochemical properties affecting oral absorption, cell uptake, protein binding, blood-brain penetration, and metabolism of bioactive substances (10). Cell membranes are relatively impermeable to hydrophilic compounds, so these are transported predominantly via paracellular route. Thus lipophilic character of the molecules enables passive diffusion through cell membrane and highly hydrophobic substances enter the cells easily. On the other hand, too high lipophilicity of drugs can be a limiting factor to oral absorption. In order to be absorbed via gut mucose, the substance needs first to be dissolved in hydrophilic mucose. Excessive lipophilicity is, thus, often linked to incomplete drug absorption after oral administration. Other mechanisms of compound transfer across the membrane not involving previous dissolution exist, such as endocytosis, but are mostly characteristic for large molecules (11). It is also generally believed that very lipophilic compounds have greater affinity for plasma-protein binding and are easily transported across the blood-brain barrier (12).

Chromatographic approach has been shown to be quite successful in modeling physicochemical and biological processes (13,14). Owing to its simplicity and efficiency, reversed-phase thin-layer chromatography appears especially attractive for lipophilicity determination (15,16). Taking into consideration that in reversed-phase chromatography solutes distribute between polar and nonpolar phases, calculated retention parameters can be adopted as indirect designators of compounds lipophilicity. 

Considering the practical importance of *s*-triazine derivatives, the main objective of this study was to examine the retention behavior of four classes synthesized *s*-triazine derivatives in reversed-phase chromatographic systems of five different mobile phases. Novelty of the paper is the correlation between *in-silico* ADME properties of s-triazine derivatives and its retention behaviour in RP-HPTLC systems.

Chromatographic data were correlated to selected physicochemical properties related to ADME properties, obtained by the established computational medicinal chemistry methods (17). Observed parameters included human intestinal absorption (HIA), plasma protein binding (PPB), blood-brain barrier (BBB) penetration, skin permeability (SP) and oral absorption (expressed as Madin-Darby canine kidney cells (MDCK) and human colorectal carcinoma cells (Caco-2) permeability). In addition, bonding affinities to different receptors (ion channel modulator (ICM), G protein-coupled receptor (GPCR) and nuclear receptor (NRL)) were estimated for studied *s*-triazine derivatives, as well as protease inhibition (PI) and kinase inhibition (KI) ability. 

Statistical validity of established correlation was tested by standard statistical parameters, such as Fisher’s criterion (*F*), correlation coefficient (*r*) and standard deviation (*s*), and *cross*-validation parameters (cross-validated coefficient of determination - *r*^2^_cv_, adjusted coefficient of determination - *r*^2^_adj_, predicted residual sum of squares - *PRESS*, total sum of squares - *TSS*, *PRESS/TSS *ratio , standard deviation based on predicted residual sum of squares - *S*_PRESS_). 

Principal component analysis (PCA), as a statistical tool for reducing dimensionality of a large number of interrelated variables and revelation of similarities among examined entities, was applied on the set of the calculated ADME properties of studied molecules. With PCA a set of new variables (principal components, PC) is defined instead of the original variables. PCs are formed by combination of the original data in such a way that the PC1 covers as much of the variation within the data set as posible. The PC2 describes the maximum amount of residual variation after the PC1 has been taken into consideration, *etc* (18). The scores plot of the two PC is a 2-D map, that provides a data overview and displays patterns or grouping within the data. The loadings plot shows relationships between variables that contribute to the positioning of the objects on the scores plot.

## Experimental

*Synthesis of s-Triazine derivatives*

The investigated compounds were 1,3,5-triazines substituted at positions 4 and 6 by smaller and larger groups with various lipophilic characteristics, chosen for investigation are presented in Table 1. Their melting points experimentaly (19, 20) and theoretical (21) observed, are shown in Table 1. All of investigated *s*-triazine derivatives were synthesized by the modified procedure of Thurston from cyanuric chloride and corresponding amines (22). In synthesis commercial cyanuric chloride (2,4,6-trichloro-1,3,5-triazine), was used (Fluka, Germany). 

**Table 1:** The chemical structures of studied s-triazines

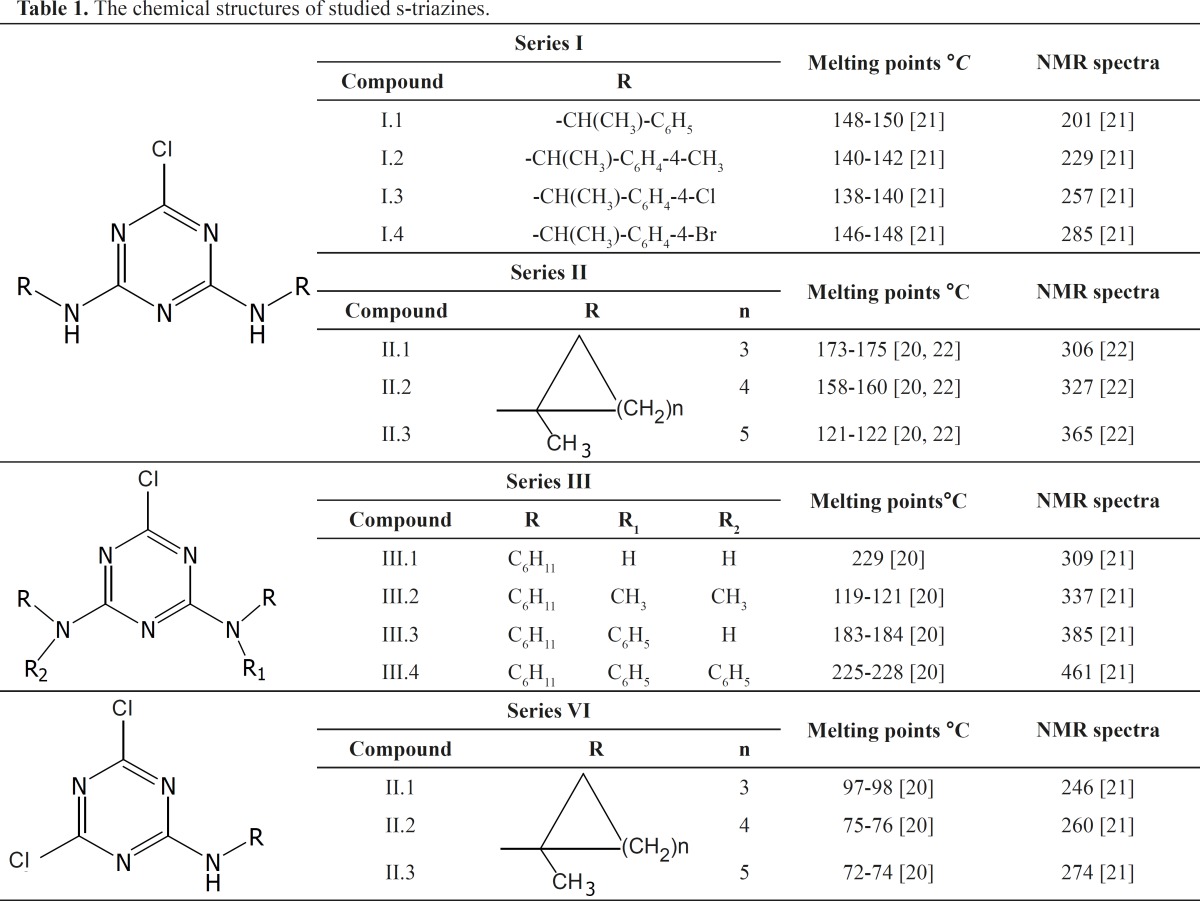

*Reversed-phase high performance thin-layer chromatography*

Precoated RP-18W/UV_254_ plates (Macherey-Nagel GmbH and Co., Düren, Germany) were used for RP HPTLC analysis. Investigated solvent mixtures used as mobile phases: acetone-water (*φ* = 0.5 - 0.8; *v/v*), acetonitrile-water (*φ* = 0.5 - 0.9; *v/v*), methanol-water (*φ* = 0.65 - 0.95; *v/v*), 2-propanol-water (*φ* = 0.4 - 0.7; *v/v*), tetrahydrofuran-water (*φ* = 0.5 - 0.75; *v/v*).

The investigated compounds were dissolved in an appropriate solvent, methanol, 1 mg mL^-1^) and the solutions (0.2 μL) were separately spotted into the plates. All the reagents used were of analytical purity. The plates were developed by the ascending technique at room temperature without previous saturation of the chamber with mobile phase. All measurements were carried out at ambient temperature. After drying of the plates, the spots were visualized under UV light at *λ* = 254 nm. *R*_F_ values were calculated as average from three measurements for each solute-mobile phase combination. For subsequent calculations mean *R*_M_ values were used; these were calculated by using the formula:

Equation (1) $$R_{M}=\log(\frac{1}{R_{F}}-1)$$

The calculated *R*_M_ values for different concentrations of organic solvent were used to check the linearity of their relationship with the volume fraction of organic modifier according to the equation (23):

Equation (2) $$R_{M}=R_{M}^{0}+\mathrm{S\emptyset}$$

Where *φ* is the volume fraction of organic solvent in the mobile phase, *R*_M_^0^ is the intercept obtained by extrapolation to *φ* = 0% of modifier, and *S* is the slope of the linear plot. Equations (1) and (2) served for deriving data for further QSAR studies.

*Statistical methods and descriptors calculation*

The complete regression analysis and PCA were carried out by PASS 2005, GESS 2006, NCSS Statistical Softwares and Statistica version 8 program (24). Physicochemical and ADME properties were calculated using the PreADMET (25) and Molinspiration online programs (26).

## Results and Discussion

***PCA***

In order to obtain some basic insight into the similarities/dissimilarities among studied molecules on the basis of their ADME properties, PCA was carried out on the set of the calculated ADME properties. The first principal component (PC1) accounted for 47.31% of data variance and the second one (PC2) for 21.41%. 

**Figure 1:**
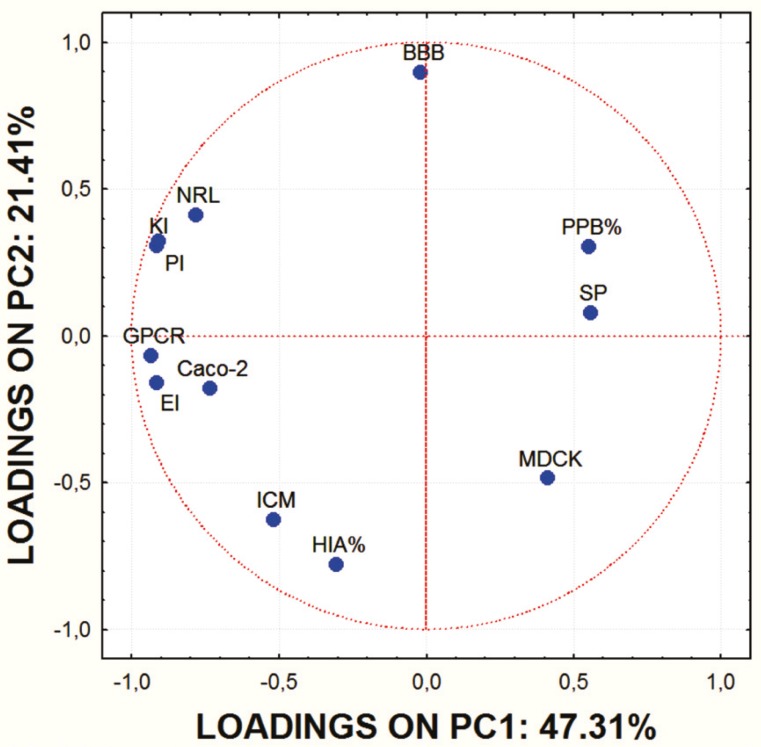
Factor loadings of ADME characteristics of examined molecules for the first two PCs.

**Figure 2:**
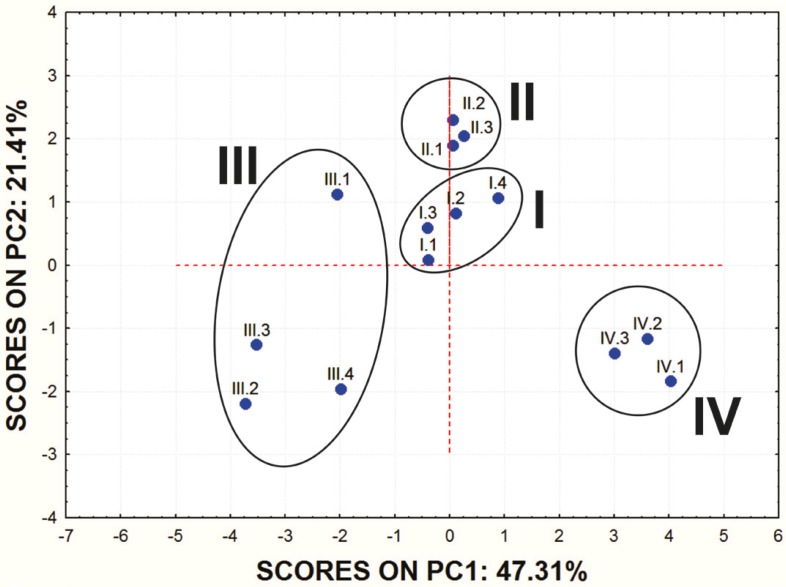
Score values of the examined molecules for the first two PCs

Score values for PC1 and PC2 are shown in Figure 1. and the mutual projections of the loading vectors in Figure 2. The loading graph reveals that significant negative influence on the PC1 have NRL, KI, PI, GPCR, EI and Caco-2 parameters. PPB, SP and MDCK have positive influence on the PC1. The most positive impact on the PC2 has BBB parameter, while ICM, HIA and MDCK parameters express negative impact on the PC2. Mentioned ADME properties are responsible for distribution of molecules on score plot that shows four well-separated groups of studied compounds. On the basis of presented PCA results it can be concluded that groups of examined molecules on the score plot are equal to the groups shown in Table 1 which are based on the molecular structure and substituents present in the examined compounds. 

Results from regression analysis using well-known equation (2) according a procedure is described earlier (27). Calculated statistics illustrate that assumed linear dependence correlates very good with experimental data. 

*Correlation of R*_M_^0^ *with biological activity predictors*

PPB, HIA, BBB, SP, MDCK and Caco-2 values (Table 2) were estimated for synthesized *s*-triazine derivatives by mathematical modeling using the PreADMET program. Molinspiration online program was used for druglikeness calculation (NRL, ICM, KI, PI, EI and GPCR). The retention data, their standard deviations and the characteristics of the $R_{M}=R_{M}^{0}=\mathrm{S\emptyset}$ equations are presented in Table 2 and Table 3. Calculated ADME parameters are presented in Table 4.

**Table 2:** The characteristics of the equations $R_{M}=R_{M}^{0}=\mathrm{S\emptyset}$ obtained for RP HPTLC separation of analysed s-triazines

**Comp.**	**methanol – water**				**2-propanol – water**			**acetone – water**				**acetonitrile – water**			**tetrahydrofuran - water**			
		***R***_M_^0^	***S***		***r***	***R***_M_^0^	***S***	***r***	***R***_M_^0^	***S***		***r***	***R***_M_^0^	***S***	***r***	***R***_M_^0^	***S***		***r***
I.1		2.457	-3.087	0.996		1.900	-3.34	0.999	3.185	-4.721	0.999		2.110	-3.501	0.998	3.439	-5.218	0.998	
I.2		3.188	-3.738	0.997		2.525	-4.196	0.993	3.580	-5.078	0.997		2.786	-4.194	0.995	3.567	-5.289	0.993	
I.3		3.976	-4.570	0.992		2.617	-4.153	0.991	3.839	-5.370	0.997		2.887	-4.215	0.996	3.538	-5.214	0.996	
I.4		4.407	-4.984	0.999		2.764	-4.332	0.993	4.303	-5.984	0.998		2.896	-4.098	0.995	3.351	-4.912	0.996	
II.1		3.181	-3.829	0.995		2.183	-3.826	0.997	3.743	-5.197	0.993		2.388	-3.347	0.997	3.289	-4.748	0.995	
II.2		3.320	-3.868	0.994		2.384	-4.024	0.998	3.935	-5.262	0.997		2.753	-3.699	0.997	3.401	-4.816	0.996	
II.3		4.530	-5.070	0.998		3.013	-4.811	0.997	4.218	-5.446	0.998		3.000	-3.788	0.995	4.071	-5.730	0.994	
III.1		3.440	-4.086	0.991		2.251	-3.962	0.996	4.118	-5.741	0.993		2.356	-3.304	0.995	3.426	-5.06	0.991	
III.2		3.998	-4.452	0.994		2.769	-4.437	0.996	4.626	-6.004	0.998		3.284	-4.197	0.998	3.611	-5.140	0.994	
III.3		3.980	-4.471	0.985		2.691	-4.383	0.988	4.019	-5.306	0.988		3.101	-4.076	0.988	3.387	-4.912	0.996	
III.4		5.295	-5.764	0.995		2.747	-4.117	0.998	4.693	-5.951	0.996		3.556	-4.394	0.997	3.902	-5.503	0.998	
IV.1		1.526	-2.206	0.999		1.118	-2.422	0.992	1.672	-2.707	0.994		1.497	-2.592	0.997	1.733	-2.897	0.996	
IV.2		1.626	-2.242	0.988		1.400	-2.910	0.991	2.034	-3.184	0.985		1.590	-2.629	0.995	1.795	-2.900	0.996	
IV.3		1.756	-2.090	0.993		1.779	-3.164	0.984	2.257	-3.286	0.995		1.956	-2.821	0.996	2.222	-3.340	0.985	

**Table 3:** Standard errors of the retention data

Molecule	HIA%	Caco-2 (nm/sec)	MDCK (nm/sec)	SP (-log*Kp*)	PPB%	BBB (C_brain_/C_blood_)	GPCR	ICM	KI	NRL	PI	EI
I.1	95.01	29.72	0.20	-2.30	89.27	4.47	0.07	-0.12	0.10	-0.20	-0.35	-0.09
I.2	95.28	31.99	0.13	-2.24	88.82	7.21	0.03	-0.17	0.06	-0.21	-0.38	-0.13
I.3	95.95	44.38	0.09	-2.22	93.61	7.94	0.06	-0.12	0.08	-0.20	-0.35	-0.10
I.4	96.22	47.80	0.02	-2.12	100.00	8.32	-0.03	-0.18	0.05	-0.29	-0.44	-0.15
II.1	92.84	24.25	1.79	-2.40	100.00	5.62	0.14	-0.19	0.01	-0.03	-0.32	-0.03
II.2	93.28	26.82	0.52	-1.95	100.00	7.36	0.15	-0.17	0.02	0.04	-0.26	-0.08
II.3	93.70	30.23	11.18	-1.59	100.00	8.75	0.15	-0.16	0.02	0.05	-0.21	-0.07
III.1	92.85	24.86	0.55	-2.99	100.00	6.62	0.23	-0.03	0.16	-0.02	-0.24	0.10
III.2	97.69	51.33	0.25	-2.58	91.59	1.21	0.34	0.02	0.12	-0.03	-0.18	0.15
III.3	97.61	52.76	1.49	-2.53	92.25	1.87	0.39	-0.08	0.26	-0.01	-0.20	0.10
III.4	98.03	53.78	20.59	-1.98	92.75	3.02	0.26	-0.07	0.10	-0.04	-0.17	0.05
IV.1	95.36	26.68	14.97	-2.16	100.00	1.44	-0.13	-0.15	-0.45	-0.53	-0.86	-0.18
IV.2	95.41	7.91	6.83	-1.99	100.00	2.10	-0.07	-0.11	-0.39	-0.39	-0.78	-0.20
IV.3	95.46	8.93	18.92	-1.83	100.00	2.99	-0.01	-0.08	-0.30	-0.30	-0.67	-0.15

**Table 4:** *In silico* ADME characteristics of studied compounds

Modifier	Acetone		Acetonitrile		Methanol		2-Propanol		Tetrahydrofuran	
Molecule	Standard error of *R*_m_^0^	Standard error of S	Standard error of *R*_m_^0^	Standard error of S	Standard error of *R*_m_^0^	Standard error of S	Standard error of *R*_m_^0^	Standard error of S	Standard error of *R*_m_^0^	Standard error of S
I. 1	0.0475	0.0705	0.7039	0.9303	0.0922	0.1106	0.1365	0.0498	0.1435	0.2276
I. 2	0.2225	0.3162	0.1381	0.1918	0.0950	0.1139	0.2608	0.1041	0.1723	0.2732
I. 3	0.1292	0.1916	0.1236	0.1717	0.1940	0.2405	0.2718	0.1085	0.1440	0.2283
I. 4	0.1341	0.1990	0.0610	0.0846	0.3027	0.3753	0.2598	0.1037	0.1133	0.1796
II. 1	0.1286	0.1828	0.1090	0.1513	0.3633	0.4355	0.1956	0.0781	0.1467	0.2326
II. 2	0.1399	0.2076	0.0967	0.1342	0.1077	0.1336	0.2893	0.1060	0.1316	0.2087
II. 3	0.2048	0.3038	0.1993	0.2767	0.0828	0.1027	0.2992	0.1194	0.1975	0.3132
III. 1	0.4059	0.6023	0.1985	0.2756	0.3306	0.4099	0.2246	0.0897	0.2856	0.4528
III. 2	0.2187	0.3246	0.1007	0.1398	0.1800	0.2231	0.2647	0.1057	0.2115	0.3353
III. 3	0.1401	0.2079	0.0901	0.1252	0.2069	0.2566	0.2537	0.1013	0.1313	0.2082
III. 4	0.2511	0.3727	0.2038	0.2830	0.1744	0.2163	0.2139	0.0854	0.1582	0.2509
IV. 1	0.1121	0.1737	0.3298	0.4579	0.4987	0.6184	0.1184	0.0472	0.2064	0.3273
IV. 2	0.1443	0.2235	0.0744	0.1033	0.0853	0.1057	0.1523	0.0608	0.1260	0.1998
IV. 3	0.3347	0.4757	0.0944	0.1311	0.2832	0.3512	0.2373	0.0947	0.1222	0.1938

Correlation analysis showed that the retention parameter correlates the best with KI and PI. Established mathematical models and its basic statistical parameters (*r*, *F*, *s*) are presented in Table 5.

**Table 5:** Polynomial correlations between retention parameter (*R*_M_^0^) and ADME descriptors of studied compounds

**Modifier**	**Dependent variable**	**Polynomial regression:** **Y* = a · *(*R***_M_^0^**)**^2^*** + b · R***_M_^0^*** + c***						**Eq** ***.***
	**Y**	***a***	***b***	***c***	***r***	***F***	***s***	
Methanol	KI	-0.0807	0.6604	-1.2173	0.9200	30.3	0.0903	3
Acetone	KI	-0.0885	0.7579	-1.5169	0.9407	42.3	0.0782	4
Tetrahydrofuran	KI	-0.1545	1.1238	-1.9476	0.9481	48.8	0.0733	5
Methanol	PI	-0.0657	0.5851	-1.5263	0.9153	28.4	0.0989	6
2-Propanol	PI	-0.1967	1.1643	-1.9652	0.9002	23.5	0.1069	7
Acetone	PI	-0.0524	0.5535	-1.6536	0.9528	54.2	0.0745	8
Acetonitrile	PI	-0.1739	1.1724	-2.1923	0.9040	24.6	0.1049	9
Tetrahydrofuran	PI	-0.0548	0.5981	-1.7104	0.9507	51.7	0.0761	10
Methanol	Caco-2	0.1015	9.8956	-1.2877	0.8045	10.0968	9.7347	11
Acetone	GPCR	0.0108	0.0520	-0.2221	0.7534	7.2244	0.1097	12
2-Propanol	NRL	-0.1709	0.9461	-1.375	0.8013	9.8696	0.1149	13
Acetonitrile	BBB%	-5.1906	26.874	-28.00	0.7433	6.7929	2.0227	14
Acetone	MDCK	5.6721	-38.794	66.88	0.6624	4.3025	6.2033	15

Correlation coefficient higher than 0.90 indicates very high correlation between *R*_M_^0^ and selected ADME properties. F-value is found statistically significant at 99% level since all the calculated *F* values are higher as compared to tabulated values. 

Equations 3-10 were cross-validated by the leave-one-out method (Table 6). High values of *r*^2^_cv_ and *r*^2^_adj_ (higher than 0.5) and *PRESS* values significantly less than *TSS* were obtained for all the models indicate that these models have very good predictive power (28). The prediction errors of the best equations (3-10) are presented in Table 7.

**Table 6:** *Cross*-validation parameters for equations 3-15

**Eq** ***.***	***r*** ^2^_cv_	***r*** ^2^_adj_	***PRESS***	***TSS***	***PRESS/TSS***	***S***_PRESS_
3	0.6543	0.8184	0.2018	0.5838	0.3457	0.1201
4	0.8261	0.8640	0.1015	0.5838	0.1739	0.0851
5	0.8418	0.8804	0.0923	0.5838	0.1581	0.0812
6	0.5995	0.8082	0.2653	0.6623	0.4006	0.1377
7	0.6916	0.7759	0.2043	0.6623	0.3085	0.1208
8	0.8703	0.8911	0.0859	0.6623	0.1297	0.0783
9	0.7295	0.7841	0.1792	0.6623	0.2706	0.1131
10	0.8678	0.8863	0.0876	0.6623	0.1323	0.0791
11	0.4387	0.5832	1659.09	2956.06	0.5613	10.8861
12	0.4132	0.4892	0.1799	0.3066	0.5867	0.1133
13	0.4486	0.5771	0.2238	0.4059	0.5514	0.1264
14	0.3017	0.4712	70.2399	100.59	0.6982	2.2398
15	-0.1577	0.3369	873.40	754.43	1.1577	7.8985

The main purpose of the conducted correlation analysis was to determine the ability to predict ADME properties of these molecules using chromatographic retention data, since the chromatography has been shown to be quite successful in modeling physicochemical and biological properties. ADME processes are dynamic in nature, as the chromatographic separations are (29). 

**Table 7:** Prediction errors of the developed equations

Molecules	Prediction errors of the developed equations							
	Eq. 3	Eq. 4	Eq. 5	Eq. 6	Eq. 7	Eq. 8	Eq. 9	Eq. 10
I. 1	0.1819	0.1008	0.0101	0.1353	0.1131	0.0723	0.1428	-0.0484
I. 2	-0.0079	-0.0021	-0.0352	-0.0513	-0.1006	-0.0364	-0.1042	-0.1058
I. 3	-0.0527	-0.0084	-0.0145	-0.1114	-0.0846	-0.0490	-0.0930	-0.0697
I. 4	-0.0758	-0.0557	-0.0333	-0.2162	-0.1902	-0.1979	-0.1845	-0.1185
II. 1	-0.,0568	-0.0700	-0.0673	0.0099	0.0409	-0.0040	0.0643	0.0160
II. 2	-0.,0657	-0.0751	-0.0674	0.0479	0.0474	0.0270	0.0227	0.0501
II. 3	-0.0983	-0.0854	-0.0469	0.0140	0.0328	0.0412	0.0302	-0.0263
III. 1	0.0605	0.0566	0.0709	0.0510	0.1010	0.0229	0.1554	0.0645
III. 2	-0.0131	0.0247	0.0241	0.0572	0.0694	0.0345	0.0376	0.0852
III. 3	0.1272	0.1604	0.1737	0.0383	0.0565	0.0755	0.0289	0.1133
III. 4	0.0831	0.0092	0.0149	0.1002	0.0812	0.0401	0.0522	0.0410
IV. 1	-0.0525	0.0471	0.0141	-0.0736	0.0494	0.0146	-0.0331	-0.0215
IV. 2	-0.0331	-0.0485	0.0382	-0.0314	-0.0593	-0.0354	-0.0122	0.0334
IV. 3	0.0065	-0.0429	-0.0867	0.0315	-0.1536	0.0013	-0.1056	-0.0180

## Conclusion

Retention constants in reverse-phase chromatography of proposed synthesized *s*-triazine derivatives have been shown to be a useful and simple way in predicting biological activity and drug likeness. For all investigated derivatives, experimentally determined retention parameters, *R*_M_^0^, could be reliably correlated with some of the ADME properties. It was found that experimentally determined retention parameter (*R*_M_^0^) of studied *s-*triazine derivatives was reliably correlated with *in-silico* calculated protease inhibition (PI) and kinase inhibition (KI) ability. Standard statistical measures and *cross*-validation parameters indicate that the established mathematical dependences between retention parametres and ADME properties are statistically valid. Also, PCA applied on both the retention parameters and calculated ADME properties showed similar grouping of molecules. That could indicate the similarity between retention behaviour and ADME properties of the examined molecules. On the basis of presented results it can be concluded that the retention parameters obtained by RP HPTLC could be successfully used for prediction of some *in-silico* ADME properties of studied compounds.
